# Mass Spectrometric Analysis of *Ehrlichia chaffeensis* Tandem Repeat Proteins Reveals Evidence of Phosphorylation and Absence of Glycosylation

**DOI:** 10.1371/journal.pone.0009552

**Published:** 2010-03-04

**Authors:** Abdul Wakeel, Xiaofeng Zhang, Jere W. McBride

**Affiliations:** 1 Department of Pathology, University of Texas Medical Branch, Galveston, Texas, United States of America; 2 Department of Microbiology and Immunology, University of Texas Medical Branch, Galveston, Texas, United States of America; 3 Center for Biodefense and Emerging Infectious Diseases, University of Texas Medical Branch, Galveston, Texas, United States of America; 4 Sealy Center for Vaccine Development, University of Texas Medical Branch, Galveston, Texas, United States of America; 5 Institute for Human Infections and Immunity, University of Texas Medical Branch, Galveston, Texas, United States of America; The Research Institute for Children at Children's Hospital New Orleans, United States of America

## Abstract

**Background:**

*Ehrlichia chaffeensis* has a small subset of immunoreactive secreted, acidic (pI ∼4), tandem repeat (TR)-containing proteins (TRPs), which exhibit abnormally large electrophoretic masses that have been associated with glycosylation of the TR domain.

**Methodology/Principal Findings:**

In this study, we examined the extent and nature of posttranslational modifications on the native TRP47 and TRP32 using mass spectrometry. Matrix-assisted laser desorption/ionization time-of-flight (MALDI-TOF) demonstrated that the mass of native TRP47 (33,104.5 Da) and TRP32 (22,736.8 Da) were slightly larger (179- and 288-Da, respectively) than their predicted masses. The anomalous migration of native and recombinant TRP47, and the recombinant TR domain (C-terminal region) were normalized by 1-ethyl-3-(3-dimethylaminopropyl)carbodiimide (EDC) modification of negatively charged carboxylates to neutral amides. Exhaustive tandem mass spectrometric analysis (92% coverage) performed on trypsin and Asp-N digested native TRP47 identified peptides consistent with their predicted masses. Two TRP47 peptides not identified were located in the normally migrating amino (N)-terminal region of TRP47 and contained predicted phosphorylation sites (tyrosine and serine residues). Moreover, native TRP47 was immunoprecipitated from *E. chaffeensis*-infected cell lysate with anti-phosphotyrosine (anti-pTyr) antibody.

**Conclusions/Significance:**

TRP47 and TRP32 are not modified by glycans and the substantial net negative charge of the ehrlichial TRPs, and particularly the highly acidic TRs present within the ehrlichial TRPs, is responsible for larger-than-predicted masses. Furthermore, this study provides evidence that the N-terminal region of the TRP47 is tyrosine phosphorylated.

## Introduction

Human monocytotropic ehrlichiosis (HME) is an emerging life-threatening tick-borne zoonosis caused by the obligately intracellular Gram-negative bacterium *Ehrlichia chaffeensis*. *E. chaffeensis* exhibits tropism for mononuclear phagocytes, and survives by evading the innate host defenses [1]–[3]. A small subset of *E. chaffeensis* proteins react strongly with antibodies in sera from infected humans or dogs [4]–[7], and the molecularly characterized immunoreactive proteins of *E. chaffeensis* include tandem repeat protein (TRP) 47, TRP120, and TRP32 (variable-length PCR target) [8]–[10]. The TR domains of the TRPs are acidic, exhibit high serine/threonine content, have predicted sites for posttranslational modifications (glycosylation and/or phosphorylation), exhibit larger-than-predicted molecular masses during electrophoresis, and contain major continuous immunodeterminants [8]–[10].

Various functions have been associated with TRPs in pathogenic bacteria, including immune evasion, adhesion, actin nucleation, and other host-pathogen interactions [11]–[18]. Similarly, TRPs identified in *E. chaffeensis* and *E. ruminantium* and closely related *Anaplasma marginale* appear to play a role in cell adhesion [19]–[23], but the function of several immunoreactive TRPs in *A. phagocytophilum* is still unknown [24]. A more recent study has demonstrated that *E. chaffeensis* TRP47 interacts with a network of host cell proteins involved in signaling, modulation of gene expression, and intracellular vesicle trafficking [25]. *E. chaffeensis* TRP47 is acidic (pI 4.2), contains seven 19-mer TRs (pI 2.9) in the C-terminal domain, and has a predicted molecular mass of 33 kDa, but exhibits an electrophoretic mass of ∼47 kDa. The TRP47 C-terminal TR domain is homologous to renin receptor, DNA polymerase III subunits gamma and tau-conserved domain, and ribonuclease E. *E. chaffeensis* TRP32 is acidic (pI, 4.1), contains four TRs, and also migrates at a larger (32 kDa) than predicted (22.5 kDa) mass.

Glycoproteins have been identified in many bacteria including *Borrelia, Chlamydia, Escherichia, Neisseria*, and *Pseudomonas* [26], [27], and many of the characterized glycoproteins appear to be involved in host-pathogen interactions [20], [26]–[30]. Moreover, carbohydrate has been detected on *Ehrlichia* and *Anaplasma* outer membrane proteins and TRPs [8], [20], [28], [31]–[35]. Glycosyltransferases have been identified in the genomes of many bacteria that have glycoproteins; however, glycosyltransferases have not been identified in *Ehrlichia* spp. genomes [36]–[38], suggesting that additional studies to define the mass of these proteins in order to understand the extent and nature of the glycans (composition, structure and attachment sites) on the native and recombinant proteins are needed.

The objective of this study was to examine the native and recombinant *E. chaffeensis* TRP47 and TRP32 using mass spectrometry (MALDI-TOF and MS/MS), in order to define the posttranslational modifications. We determined by mass spectrometry that the native TRP47 and TRP32 were nearly identical to the predicted mass. Furthermore, we demonstrate that the highly acidic TRs present within the ehrlichial TRPs are responsible for the anomalous electrophoretic behavior of these proteins and not glycosylation. Moreover, we provide mass spectrometry and immunoprecipitation evidence that TRP47 is tyrosine phosphorylated.

## Results

### Analysis of *E. chaffeensis* Secreted Proteins by Single and Two-Dimensional Gel Electrophoresis (2-DE) and Western Immunoblotting

**Figure 1:**
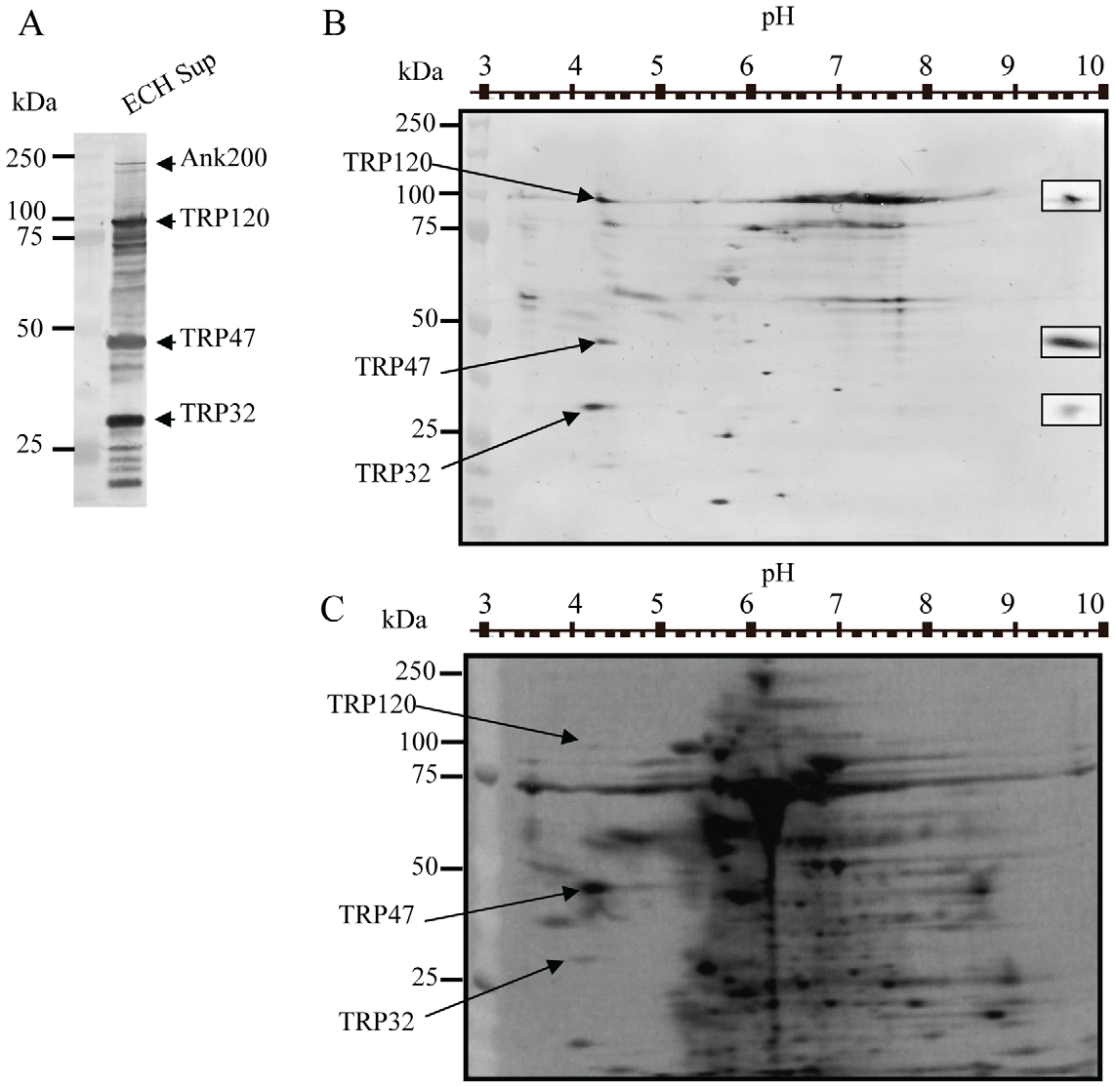
Separation and purification of *E. chaffeensis* secreted major immunoreactive proteins by one-dimensional and two-dimensional gel electrophoresis (2-DE). (**A**) Cell-free supernatant collected from *E. chaffeensis* infected DH82 cells was precipitated with 20% ammonium sulfate before separation by SDS-PAGE. The TRP32, TRP47, TRP120, and Ank200 were major immunoreactive proteins as determined by Western immunoblotting with canine anti-*E. chaffeensis* serum. (**B**) Western immunoblot and (**C**) silver stained gel of *E. chaffeensis-*secreted proteins collected from cell-free *E. chaffeensis-*infected DH82 cells resolved by 2-DE. The approximate pH and molecular mass standards of the proteins are shown on top and left side of the Western immunoblot and silver stained gel images. The *E. chaffeensis* 2-DE gel resolved proteins were detected by anti-*E. chaffeensis* serum. The arrow indicates the three acidic, major immunoreactive proteins separated by 2-DE according to their respective pI and larger-than-the-predicted molecular mass (TRP120, TRP47, and TRP32). TRP120, −47, and −32 spots detected with protein-specific antibodies are shown as insets, from top to bottom on right side of the image (B).

Examination of the *E. chaffeensis-*secreted proteome by Western immunoblotting using dog anti*–E. chaffeensis* identified several major immunoreactive proteins (Figure 1A). The highly acidic TRPs proteins, including TRP120 (pI 4.1), TRP47 (pI 4.2), and TRP32 (pI 4.1), which were distinctly separated and resolved during 2-DE, were clearly visible on the left side of the immunoblot forming a column at positions corresponding to their pIs (between 4.0 and 4.5) and molecular masses. All of these proteins migrated at larger-than their predicted molecular masses, ∼100-, 47- and 32-kDa, respectively (Figures 1B and 1C). Each of these proteins was identified with TRP-specific antibodies (see insets Figure 1B). The TRP47 and TRP32 were examined further to define the posttranslational modifications.

### MALDI-TOF Mass Spectrometric Analysis of Native and Recombinant TRPs

**Figure 2:**
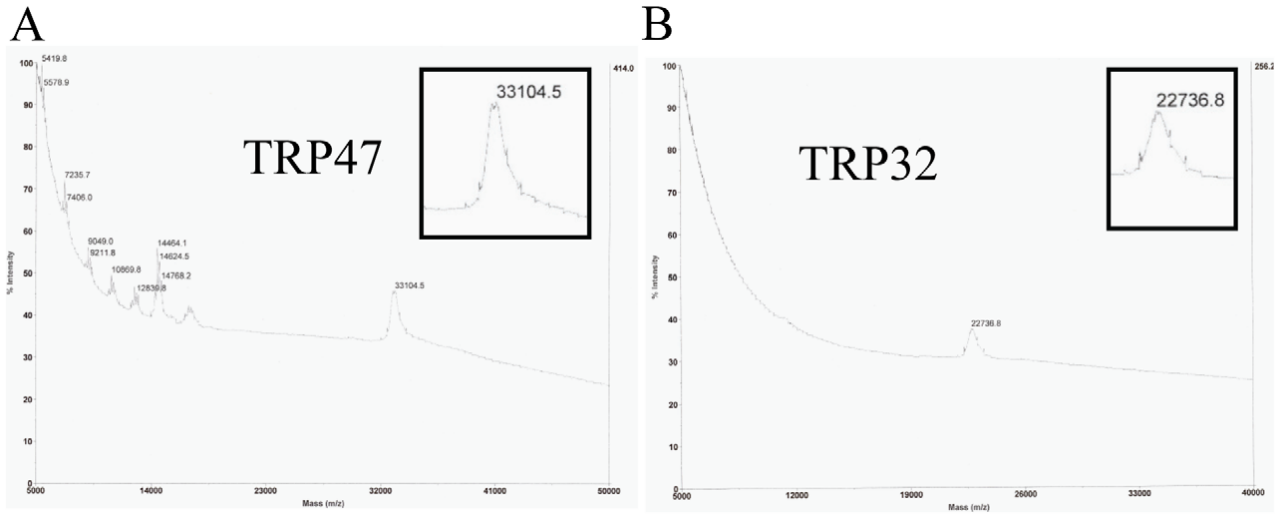
MALDI-TOF mass spectra of *E. chaffeensis* native TRP47 and native TRP32. (**A**) For native TRP47, the mass-spectrum was recorded within the range of m/z 5,000 to 50,000 illustrates a peak of TRP47 singly charged ion (33,104.5). (**B**) For native TRP32, the mass-spectrum recorded within the range of m/z 5,000 to 40,000 illustrates a peak of the TRP32 singly charged ion (22,736.8). The relative intensities of the ions are shown on the *y* axis; the mass to charge ratios are shown on the *x* axis.

We determined the mass of the native TRP32 and TRP47 purified from 2-DE by MALDI-TOF mass spectrometry. For native *E. chaffeensis* TRP47, the mass spectrum was recorded within the range of m/z 5,000 to 50,000 and a singly charged ion was recorded at 33,104.5 m/z demonstrating the molecular mass of native TRP47 was 179 Da larger than the predicted molecular mass of 32,925 Da (Figure 2A and Table 1). The mass spectrum of native TRP32 was recorded within the m/z range of 5,000 to 40,000. A singly charged ion was recorded at 22,736.8 m/z demonstrating that native TRP32 was 288 Da larger than the predicted molecular mass of 22,449 Da (Figure 2B and Table 1). We also examined the recombinant TRP47 proteins GST-TRP47 and GST-CterTRP47 that also exhibited larger than their predicted molecular masses by SDS-PAGE. The recombinant GST-TRP47 and GST-CterTRP47 molecular mass as determined by MALDI-TOF was 56,698 Da (predicted 56,581) and 43,522 Da (predicted 43,525), respectively, and close to the predicted masses demonstrating that these polypeptides were not modified (Table 1).

**Table 1:** Molecular mass determinations of *E. chaffeensis* TRP47 and TRP32 by MALDI-TOF.

*E. chaffeensis* protein	OM (Da)	PM (Da)	MS (Da)	Mass difference (OM-PM in Da)	Mass difference (PM-MS in Da)
Native TRP47	∼47,000	32,925.5	33,104.5	14,075	179
Native TRP32	∼32,000	22,449.0	22,736.8	9,550	288
GST-TRP47	∼67,000	56,581.6	56,698.0	10,418	116
GST-NterTRP47	∼42,000	41,580.6	ND	419	ND
GST-CterTRP47	∼55,000	43,525.4	43,522.0	11,474	3

OM, Observed molecular mass in SDS-PAGE; PM, Predicted molecular mass; MS, molecular mass as determined by MALDI-TOF mass spectrometry; Da, Dalton; and ND, not determined.

### MALDI-MS and Tandem (MS/MS) Mass Spectrometric Analysis of Trypsin-Digested TRP47

MALDI-MS performed on trypsin-digested TRP47 peptides exhibited high relative intensity of several abundant ions (m/z). Those abundant ions with high relative intensity were selected for further MS/MS analysis. For protein/peptide identification, the bacteria NCBI endopeptidase taxonomy searched in the NCBI non redundant database identified three peptides with significant protein/peptide match, based on both the peptide mass fingerprint (PMF) and the MS/MS data from several precursor ions with low expectation values and high protein score. A total of 12% sequence coverage was achieved with trypsin digested TRP47, which included three of the nine peptides that were generated from typsin-digestion (Table 2). The identified peptides were NNGHVISDFR, GVQAENFVFDIK, and DSLLNEEDMAAQFGNR with molecular masses of 1158.2, 1366.5, and 1809.9 Da, respectively, and consistent with the predicted molecular masses indicating they were not posttranslationally modified. The remaining six peptides were not identified by MS/MS analysis of TRP47 tryptic digests.

**Table 2:** The list of the predicted trypsin digested native TRP47 peptides from *E. chaffeensis*.

Position of cleavage site	Peptide sequence	Peptide length [aa]	Peptide mass [Da]
24	MLHLTTEINDIDFSNNLNIYSGNR	24	2,795.1
49	FVVTSGDMQVDVGSEPDHGYHILFK	25	2,778.1
59	**NNGHVISDFR**	10	1,158.2
71	**GVQAENFVFDIK**	12	1,366.5
76	NHNLR	5	652.7
125	ASFLVDPMAPFTELDNSQHPHFVVNMHTANECGSDCVHHNEHDHDAHGR	49	5,504.9
296	GAASSVAEGVGSAISQILSLSDSIVVPVLEGN*ASVSEGDAVVNAVSQEAPAASVSEGDAVVNAVSQETPAASVSEGDAVVNAVSQETPAASVSEGDAVVNAVSQETPAASVSEGDAVVNAVSQETPAASVSEGDAVVNAVSQETPAASVSEGDAVVNAVSQETPA*TQPQSR	171	16,385.4
312	**DSLLNEEDMAAQFGNR**	16	1,809.9
316	YFYF	4	638.7

The identified peptides are shown in bold letters and TR-containing region is italicized. aa, amino acids; Da, Dalton.

### MALDI-MS and Tandem Mass Spectrometric Analysis of Asp-N Digested TRP47

To obtain more sequence coverage, TRP47 was digested with Asp-N endopeptidase resulting in 23 peptide fragments. Twenty-one peptides with molecular masses were identified extending the total TRP47 sequence coverage to 90%, and these peptides all had masses that matched their predicted molecular masses (Table 3). The acidic TRs of TRP47, which exhibited abnormal electrophoretic mobility and contained predicted sites of posttranslational modifications (phosphorylation/glycosylation) were also identified. Their molecular masses were consistent with their predicted molecular masses demonstrating absence of posttranslational modifications. Two unidentified peptides (DHG**Y**HILFKNNGHVIS and DSIVVPVLEGNA**S**V**S**EG) with predicted molecular masses of 1851.0 and 1671.8 Da, respectively, were located in the normally migrating amino-terminal part of TRP47 (outside the TR containing region of TRP47). The peptide DHG**Y**HILFKNNGHVIS contains a single tyrosine (Y44), and peptide DSIVVPVLEGNA**S**V**S**EG has three serine residues (S148, S159, S161), and NetPhos 2.0 prediction server identified Y44, S159, and S161 as phosphorylation sites (Table 3). In addition, phosphopeptides were not detected by analysis with ESI-LC MS/MS and MALDI MS/MS on phosphopeptide-enriched TRP47 trypsin and Asp-N digests.

**Table 3:** The list of the predicted Asp-N endoproteinase digested native TRP47 peptides from *E. chaffeensis*.

Position of cleavage site	Peptide sequence	Peptide length [aa]	Peptide mass [Da]
9	**MLHLTTEIN**	9	1,071.3
11	**DI**	2	246.3
30	**DFSNNLNIYSGNRFVVTSG**	19	2,104.3
34	**DMQV**	4	491.6
40	**DVGSEP**	6	602.6
56	DHGYHILFKNNGHVIS	16	1,851.1
68	**DFRGVQAENFVF**	12	1,428.6
81	**DIKNHNLRASFLV**	13	1,526.8
90	**DPMAPFTEL**	9	1,020.2
110	**DNSQHPHFVVNMHTANECGS [carbamidomethyl C(18)]**	20	2,224.4 [2,280.9]
118	**DCVHHNEH [carbamidomethyl C(2)]**	8	990.0 [1,047.5]
120	**DH**	2	270.2
146	**DAHGRGAASSVAEGVGSAISQILSLS**	26	2,440.7
163	DSIVVPVLEGNASVSEG	17	1,671.8
182	**DAVVNAVSQEAPAASVSEG** (TR)	19	1,800.9
201	**DAVVNAVSQETPAASVSEG** (TR)	19	1,830.9
220	**DAVVNAVSQETPAASVSEG** (TR)	19	1,830.9
239	**DAVVNAVSQETPAASVSEG** (TR)	19	1,830.9
258	**DAVVNAVSQETPAASVSEG** (TR)	19	1,830.9
277	**DAVVNAVSQETPAASVSEG** (TR)	19	1,830.9
296	**DAVVNAVSQETPATQPQSR** (TR)	19	1,998.1
303	**DSLLNEE**	7	818.8
316	**DMAAQFGNRYFYF**	13	1,629.8

The identified peptides are shown in bold letters. aa, amino acids; Da, Dalton; and (TR), tandem repeat.

### Immunoprecipitation of TRP47 with Anti-Phosphotyrosine (Anti-pTyr) Antibody

**Figure 3:**
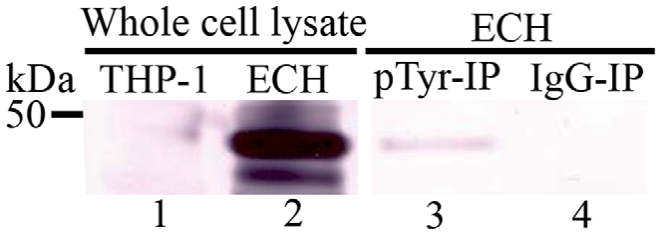
Immunoprecipitation of TRP47 with anti-pTyr antibody. Whole cell lysates from uninfected (THP-1) and *E. chaffeensis-*infected THP-1 cells (ECH) probed with anti-TRP47 antibody [lanes 1 and 2]. ECH whole cell lysates immunoprecipitated with, mouse anti-pTyr antibody (pTyr-IP, lane 3), normal mouse IgG (IgG-IP, lane 4) and detected with TRP47 antibody.

We have previously [25] demonstrated that TRP47 physically interacts with protein tyrosine kinase, FYN and genes encoding protein tyrosine kinases are not present in the *E. chaffeensis* genome [36]. Based on our present MALDI-TOF analysis of native TRP47 (small mass difference suggesting phosphorylation and not glycosylation) and previous observations, we hypothesized that upon *E. chaffensis* interaction with the host cell, TRP47 is phosphorylated by a host cell kinase. To test this hypothesis, we immunoprecipitated proteins from *E. chaffeensis-* infected THP-1 cells with anti-pTyr and detected the immunoprecipitated proteins with TRP47 antibody. TRP47 was detected only in *E. chaffeensis-* infected cell lysate precipitated with anti-pTyr, but not with normal mouse IgG (Figure 3).

### Chemical Modification of Native and Recombinant TRP47

**Figure 4:**
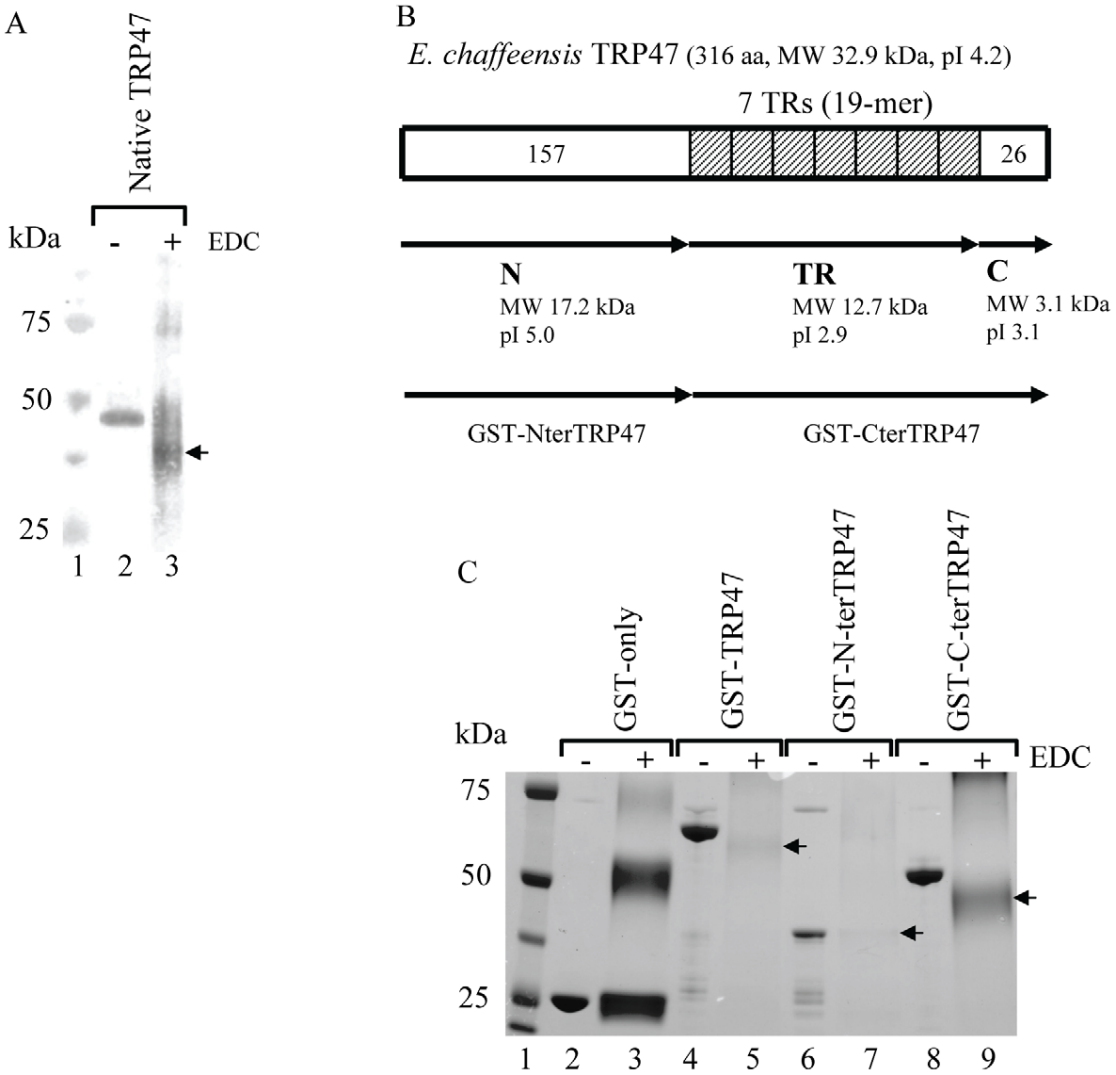
EDC modification of native and recombinant *E. chaffeensis* TRP47. (**A**) Western immunoblot of native TRP47 detected with rabbit anti-TRP47 serum. Lane 1, molecular mass standards; lane 2, unmodified native TRP47 (2.5 µg); lane 3, EDC-modified TRP47 (2.5 µg). (**B**) Schematic representation of TRP47 showing amino-terminal (N), tandem repeats (TR), and carboxy-terminal (C) regions with predicted molecular weight and pI of native protein, also represented are the recombinant GST-NterTRP47 and GST-CterTRP47. (**C**) Coomassie blue staining of proteins resolved by SDS-PAGE, 1, molecular mass standards; lane 2, unmodified GST-only (2.5 µg); lane 3, EDC-modified GST-only (2.5 µg); lane 4, unmodified GST-TRP47 (2.5 µg); lane 5, EDC-modified GST-TRP47 (2.5 µg); lane 6, unmodified GST-NterTRP47 (1.5 µg); lane 7, EDC-modified GST-NterTRP47 (1.5 µg); lane 8, unmodified GST-CterTRP47 (2.5 µg); lane 9, EDC-modified GST-CterTRP47 in excess of ethanolamine. (−), unmodified; (+), EDC (1-ethyl-3-(3-dimethylaminopropyl)carbodiimide)-modified; arrow, indicates the EDC modified protein band; aa, amino acids; MW, molecular weight; and pI, isoelectric potential.

*E. chaffeensis* TRP47 is a 316 amino acid, acidic (pI, 4.2), secreted, protein containing seven 19-mer TRs (ASVSEGDAVVNAVSQETPA) that encompasses the majority of the carboxy (C)-terminal portion of the protein. The TR domain is more acidic (pI 2.9) than the amino (N)-terminal region (pI 5.0) (Figure 4B). Based on the amino acid sequence, TRP47 contains a typical percentage (15%) of acidic residues, but a much lower (3%) percentage of basic residues (Table 4). To demonstrate that the acidic nature of the protein contributed substantially to the abnormal electrophoretic behavior, we chemically modified native TRP47 and recombinant GST-TRP47, GST-NterTRP47, and GST-CterTRP47 with EDC in the presence of an excess of an amine which converts negatively charged carboxylates into neutral amides (by neutralizing negatively charged acidic residues). EDC-modified native TRP47, GST-TRP47, and GST-CterTRP47 migrated faster at about 37±3 kDa, 60±3 kDa, and 46±3 kDa, close to their true molecular masses, than the unmodified proteins that migrate at 47 kDa, 67 kDa, and 55 kDa in SDS-PAGE gel (Figures 4A, 4C and Table 4). However, the migration of the less acidic proteins such as GST-only (pI 5.9) and GST-NterTRP47 (pI 5.5) were unchanged after EDC treatment, suggesting numerous acidic residues present on TRP47 and C-terminal TRP47 (GST-CterTRP47) are responsible to a large extent for the anomalous migration of these proteins on SDS-PAGE (Figure 4C and Table 4). EDC-modification of native and recombinant TRP47 resulted in much more diffused electrophoretic bands and dimers and trimers representing intermolecular cross-linkages were observed (Figures 4A and 4C).

**Table 4:** Molecular characteristics of native TRP47 and recombinant full-length, N- and C-terminal fragments.

Protein	Molecular mass (kDa)			pI	No. (%) of acidic residues^1^	No. (%) of basic residues^2^	Ratio of acidic to basic residues (mass difference observed after EDC treatment in kDa)
	Predicted	SDS-PAGE	EDC				
Native TRP47	32.9	47	37	4.18	46 (14.5)	8 (2.53)	5.7 (10)
GST-TRP47	56.6	67	60	4.64	78 (14.9)	37 (7.08)	2.1 (7)
GST-NterTRP47	41.6	42	42	5.47	53 (14.4)	36 (9.78)	1.5 (0)
GST-CterTRP47	43.5	55	46	4.48	62 (15.4)	32 (7.96)	1.9 (9)

EDC, molecular mass post 1-ethyl-3-(3-dimethylaminopropyl)-carbodiimide treatment in SDS-PAGE; ^1^, aspartic acid + glutamic acid; and ^2^, arginine + lysine.

## Discussion

The anomalous migration of *Ehrlichia* and *Anaplasma* TRPs has been reported in numerous studies [8], [9], [24], [31], [32]. Furthermore, native and recombinant *Ehrlichia* TRPs exhibit nearly identical larger than predicted molecular masses, suggesting that the native and recombinant proteins have similar properties and modifications. The basis of the anomalous migration of TRPs had been previously associated with posttranslational glycosylation, particularly by O-linked glycosylation of *Ehrlichia* TRPs based primarily on the larger than predicted molecular masses, detection of carbohydrate on recombinant TRP proteins, the high proportion of serine/threonine residues (O-linked glycosylation sites), similarity to other O-glycosylated (mucin-like) proteins, and predictions (YinOYang 1.2, NetOGlyc 3.1) that identified potential O-linked glycosylation sites [8], [31], [32]. Previous studies have clearly demonstrated that many pathogenic bacteria such as *Neisseria meningitidis*, *Neisseria gonorrhoeae*, *Pseudomonas aeruginosa*, *Helicobacter pylori*, and *Campylobacter coli* are capable of O-linked protein glycosylation with a wide variety of carbohydrates [39]–[43], and glycosylated proteins exhibit an abnormal migration in SDS-PAGE [26]. However, mass spectrometry had not been performed to unequivocally determine the mass of the native or recombinant *Ehrlichia* TRPs and the exact nature of the posttranslational modifications and the glycan attachment sites.

In this investigation, we examined two molecularly characterized *E. chaffeensis* TRPs in order to fully understand the nature of posttranslational modifications associated with these proteins. A primary goal of this study was to examine the native ehrlichial proteins, so that differences in native and recombinant protein modifications could be determined. MALDI-TOF demonstrated that the masses of native TRP47 and TRP32 were slightly larger (179- and 288-Da, repectively) than their predicted masses, and too small to account for glycan modification. Similarly, the recombinant TRP47 fragments had masses that were consistent with their predicted masses, demonstrating that glycan modifications were not present. We have also reported similar observations with regard to other recombinant ehrlichial TRPs, including TRP32 and TRP120 that exhibited larger than predicted masses [9], [44].

We also investigated the small mass difference of the native TRP47 to explore the possibility of phosphorylation. Exhaustive MS/MS of the native TRP47 identified all peptides except two associated with the N-terminal region of the protein that also contained predicted phosphorylation sites (NetPhos 2.0). Protein phosphorylation is one of the important posttranslational modification processes reported on effector proteins of other obligately intracellular organisms such as *Chlamydia trachomatis* and *A. phagocytophilum* [45]–[49]. Thus, the inability to detect these peptides could be related to phosphorylation, which results in a lower level of ionization, and a characteristic of phosphorylated peptides. Identifying phosphorylation sites using mass spectrometry has proved to be a challenging task particularly when there are very few phosphorylated peptides present in the digested sample [50], [51].

In our previous study, we have demonstrated that TRP47 is present on the surface of dense-cored ehrlichiae and interacts with the host tyrosine kinase FYN [25]. In this present study, we demonstrated by immunoprecipitation with anti-pTyr antibody that TRP47 is tyrosine phosphorylated, presumably by a host cell tyrosine kinase. Two peptides unidentified by MS/MS were 41-DHG**Y**HILFK-49 and 147-DSIVVPVLEGNA**S**V**S**EG-163, which contain predicted phosphorylation sites (Y44, S148, S159, S161). As discussed earlier, phosphorylated peptides do not ionize efficiently and are often undetected by mass spectrometry, and thus could explain our inability to identify these peptides by this method [50], [51]. Immunoprecipitaion of TRP47 with anti-pTyr antibody provides evidence that this protein is phosphorylated and the MS-MS results confirmed that all peptides identified were unmodified, except two containing potential phosphorylation sites. NetPhos prediction indicated that most probable TRP47 phosphorylated residues were **Y**44 (peptide 41–49) and **S**159 and/or **S**161 (peptide 147–163). The small molecular mass differences between the predicted and MALDI-TOF and MS/MS observed for native TRP47 (179 Da) and TRP32 (288 Da) suggest that the mass of the modifications is consistent with two or three phosphates outside the TR region, and confirmed that the abnormally migrating TR region is not modified by glycans or phosphate.

We determined that the abnormal electrophoretic masses of the TRP47 were associated with the highly acidic TR domain, suggesting that the abnormal migration is due to acidic nature of the TRs within these ehrlichial TRPs [9], [44]. All of the characterized *Ehrlichia* TRPs that exhibit abnormal electrophoretic masses are acidic, and this biophysical property has been associated with abnormal electrophoretic migration of other acidic proteins such as caldesmon, HPV 16 E7, ribonuclease U2, and Gir2 [52]–[55]. EDC-modified native TRP47, recombinant GST-TRP47 and GST-CterTRP47 having a substantially higher number of acidic residues compared to the basic residues (ratio of 2∶1 to 6∶1) migrated much faster than the unmodified protein, close to their predicted molecular masses as observed by others [52]. The migration of GST and GST-NterTRP47 having similar numbers of acidic and basic residues (the ratio of acidic/basic residues between 1-1.5∶1) did not change after EDC modification. The EDC-modified protein exhibited a more diffused electrophoretic band than that of unmodified protein, an effect that could be due to different levels of esterification and some EDC-mediated intermolecular cross-linkage of proteins was observed, which is consistent with previous reports [52]–[55]. Moreover, compared to Gir2, which is an intrinsically unstructured protein, TRP47 is a mostly folded and ordered protein [56]. Thus, the abnormal migration of these acidic TRPs appears to be related to the presence of higher ratio of acidic (primarily Asp+Glu) to basic residues (Arg+Lys) as demonstrated by their near normal electrophoretic behavior after chemical modification of native and recombinant TRP47 with EDC [56]. It has been proposed that acidic domains have incomplete SDS binding [55], [56].

In conclusion, the results of three independent analyses, experiments based on mass spectrometry and those based on immunoprecipitation and chemical modification, support the conclusion that the TR region of TRP47 is not glycosylated; however, the protein appears to be phosphorylated in the N-terminal region preceeding the TRs. A recent study [57] of several *Borrelia burgdorferi* proteins such as FlaA, FlaB, OspA, and OspB previously reported to be glycosylated found no evidence of glycosylation by mass spectrometric analysis. Some of the small mass difference observed for TRP47 and TRP32 could be attributed to carbamidomethylation of cysteine (C, 57 Da) and oxidation of methionine residue (M, 16 Da) during sample preparation. Bioinformatic analysis of *Ehrlichia* genomes has revealed no evidence of conserved glycosyltransferases, enzymes that catalyzes the transfer of an activated donor sugar to an appropriate acceptor, typically another sugar, lipid, protein or small molecule [36]–[38]. Still, we cannot rule out the possibility of the existence of a novel glycosyltransferase in the genome of *E. chaffeensis*. Moreover, others have reported glycosylation on outer membrane proteins (omp1/p28 and p44/msp2, respectively) of *Ehrlichia* and *Anaplasma* [28], [33]–[35]. However, this study has defined the molecular basis for the anomalous electrophoretic migration of immunoreactive, acidic *E. chaffeensis* TRPs, and determined these proteins are not glycosylated.

## Materials and Methods

### Cultivation of *E. chaffeensis*

Cultivation of ehrlichiae was performed in DH82 cells as previously described [31]. Cell culture supernatant was collected from 95–100% *E. chaffeensis* (Arkansas)*-*infected DH82 cells maintained in serum-free media supplemented with 1% HEPES buffer, 1% sodium pyruvate and 1% non-essential amino acids at 37°C in a humidified 5% CO_2_ atmosphere. Normal and *E. chaffeensis-*infected THP-1 cells were cultured as described [25]. The level of ehrlichial infection was assessed by Diff-Quik staining. The cell culture supernatant harvested was centrifuged at 10,000×*g* for 10 min, and was used immediately or frozen at −80°C until further use.

### Protein Sample Preparation for 2-DE

For 2-DE sample preparation was performed according to the manufacturer's protocol (Invitrogen, Carlsbad, CA) with slight modifications. Briefly, the cell-free supernatant collected from *E. chaffeensis-*infected DH82 cells was concentrated 50 times using Amicon Ultra-15 Centrifugal Filter Unit with Ultracel-10 membrane (Millipore, Billerica, MA.) and desalted with a Zeba spin column (Pierce, Rockford, IL). To 100 µl of desalted protein, 1 µl Tris Base (1 M, Biorad, Hercules, CA), 1 µl 100X Protease inhibitor cocktail (Pierce), and 1 µl dithiothreitol (DTT 2 M, Sigma Chemical, Saint Louis, MI) were added, mixed and the pH was adjusted between 8.4–9.0. The sample was then treated with 100 units of Benzonase (Sigma) and 0.5 µl of N,N-dimethylacrylamide (DMA, Sigma) each for 30 min at room temperature. The excess DMA was quenched by adding 1 µl DTT, and the mixture was centrifuged at 16,000×g for 20 min at 4°C.

### 2-DE and Western Immunoblotting

To 110 µg of protein sample (10 mg/ml), 128 µl of 1.1x ZOOM 2D protein solubilizer 1, 0.7 µl DTT (2 M), 0.3 µl Bio-Lyte 3/10 carrier ampholyte, and trace amounts of Bromophenol Blue were added, mixed and rehydrated onto a ZOOM strip (pH 3–10) for 1 h at room temperature. The protein on the strip was resolved in the first dimension by isoelectric focusing (IEF) in the ZOOM IPGRunner mini-cell following the manufacturer's protocol (Invitrogen) at 4000 Vh. Following focusing, ZOOM strips were equilibrated in 10 ml of buffer containing 1X NuPAGE LDS sample buffer and 1X NuPAGE sample reducing agent, and then equilibrated in 10 ml of 1X NuPAGE LDS sample buffer with 125 mM iodoacetamide sequentially for 15 min each. After equilibration, the IPG strips were transferred to the top of a 4–12% Bis-Tris Zoom SDS-polyacrylamide gradient gel (Invitrogen) and overlaid with 0.5% agarose in Laemmli buffer for second-dimension SDS-polyacrylamide gel electrophoresis. Following electrophoresis, proteins were detected with ProteoSilver Plus Silver Stain Kit (Sigma), Zinc Stain (Biorad, Hercules, CA), or transferred to nitrocellulose membrane and detected with antibodies specific for TRP32, TRP47, TRP120 or *E. chaffeensis* as previously described [8], [9], [44].

### Cloning and Expression of Recombinant *E. chaffeensis* TRP47

For recombinant TRP47, in-frame GST fusion proteins for full-length TRP47, amino (N)-terminal (TRP47_1–380_) and carboxy (C)-terminal (TRP47_361–842_) TRP47 were generated by PCR, amplifying the corresponding coding regions from *E. chaffeensis* Arkansas genomic DNA using custom synthesized oligonucleotide primers [25]. pGEX-6P-1 plasmids encoding the GST-TRP47, GST-TRP47_1–380_ (GST-NterTRP47), and GST-TRP47_361–842_ (GST-CterTRP47) fusion proteins were transformed into BL21 strain of *Escherichia coli* (GE Healthcare Bio-Sciences Corp., Piscataway, NJ). Protein expression and purification were performed according to the procedures outlined in the Bulk GST Purification Module (GE Healthcare).

### Sample Preparation for Mass Spectrometry

The protein of interest was excised from the 2-DE gel with a clean scalpel and placed into a microcentrifuge tube and destained following the manufacturer's protocol. Briefly, the gel slices were cut into equal 1 mm pieces, and proteins were electroeluted in volatile buffer (50 mM ammonium bicarbonate with 0.025% SDS) using D-Tube Dialyzers (Novagen-EMD Biosciences, San Diego, CA). The eluted protein was desalted and concentrated using Amicon-Ultra 4 (Millipore), and the amount and purity was verified by Coomassie staining and Western immunoblotting using TRP47- and TRP32-specific serum before proceeding for mass spectrometry analysis. For in-gel digestion of protein isolated by 2-DE, the proteins of interest were excised from the gel, destained, and digested with trypsin (Sigma) and Asp-N endoproteinase (Sigma) following the manufacturer's protocol (Sigma). After digestion, the samples were removed from the incubator, and 1 µl of sample solution was spotted directly onto a MALDI target plate and allowed to air dry. 1 µl of alpha-cyano-4-hydroxycinnamic acid (for digested peptides) or sinapic acid (for undigested protein) (Aldrich, Milwaukee, WI) matrix solution (50∶50 acetonitrile/water at 5 mg/mL) was then applied on the sample spot and allowed to dry. The dried MALDI spot was blown with compressed air (Decon Laboratories, King of Prussia, PA) before inserting into the mass spectrometer. To detect phosphopeptide, enrichment from protein digests was performed using a phosphopeptide isolation kit (Pierce) and the samples were analyzed by electrospray (ESI) LC-MS and MALDI-TOF spectrometers following the manufacturer's protocol (Pierce).

### Mass Spectrometry

Matrix-assisted laser desorption/ionization time-of-flight mass spectrometry (MALDI TOF-MS) was used to analyze the mass of the native TRP47, TRP32 and recombinant TRP47 as previously described [44]. Data were acquired with an Applied Biosystems 4800 MALDI-TOF/TOF Proteomic Analyzer. Applied Biosystems software packages including 4000 Series Explorer (v3.6 RC1) with Oracle Database Schema Version (v3.19.0) and Data Version (3.80.0) were used to acquire both MS and MS/MS data. The instrument was operated in positive ion linear mode, with mass range as required. A total 4,000 laser shots were acquired and averaged from each sample spot. External calibration was performed using cytochrome *c* or bovine serum albumin according to the target molecular weight. MALDI TOF/TOF mass spectrometry was performed for peptide mass fingerprinting (PMF) as described [58] with some modifications. The instrument was operated in positive ion reflectron mode in 850–3,000 Da mass range, and the focus mass was set at 1,700 Da. For MS data, 2,000–4,000 laser shots were acquired and averaged from each sample spot. Automatic external calibration was performed using a peptide mixture with reference masses 904.468, 1296.685, 1570.677, and 2465.199 Da. Following MALDI MS analysis, MALDI MS/MS was performed on several (5–10) abundant ions from each sample spot. A 1 kV positive ion MS/MS method was used to acquire data under post-source decay (PSD) conditions with precursor selection window set at +/− 3 Da. For MS/MS data, 2,000 laser shots were acquired and averaged from each sample spot. Automatic external calibration was performed using reference fragment molecular masses 175.120, 480.257, 684.347, 1056.475, and 1441.635 (from precursor mass 1570.677). Alpha- casein digested with trypsin was used as control for phophopeptide identification. Applied Biosystems GPS Explorer (v3.6) software was used in conjunction with MASCOT to search the respective protein database (NCBI) using both MS and MS/MS spectral data for protein identification. Protein match probabilities were determined using expectation values and/or MASCOT protein scores. MS peak filtering included the following parameters, mass range 800–4,000 Da, minimum S/N filter 10, mass exclusion list tolerance 0.5 Da, and mass exclusion list included 842.51, 870.45, 1045.56, 1179.60, 1277.71, 1475.79, and 2211.10 (trypsin and keratin fragments). Other parameters included were the following: selecting enzyme as trypsin or Asp-N; maximum missed cleavages  = 1; fixed modifications included carbamidomethyl (C); variable modifications included oxidation (M); precursor tolerance set at 0.2 Da; MS/MS fragment tolerance set at 0.3 Da; mass  =  monoisotopic; and peptide charges were only considered as +1. The significance of a protein match based on both the PMF in the first MS and the MS/MS from several precursor ions, is based on expectation values. The default significance threshold was p<0.05. A more stringent threshold of 0.001 was used for protein identification.

### Antibodies

Rabbit anti-TRP47, -TRP32, and -TRP120 antibodies were produced to KLH-conjugated peptides representing major continuous epitopes as previously described [8], [9], [44]. Convalescent-phase anti-*E. chaffeensis* dog serum was obtained from an experimentally infected dog (no. 2251) [8]. Other antibodies used in this study were mouse anti-pTyr (PY99) and normal mouse IgG (Santa Cruz Biotechnology, Santa Cruz, CA).

### Coimmunoprecipitation

Immunoprecipitation was performed as described [25] with modifications. Briefly, 10^7^ normal and *E. chaffeensis-*infected THP-1 cells were collected (500×g, 5 min), washed twice in ice-cold phosphate buffered saline (PBS), resuspended in 1 ml of ice-cold RIPA lysis buffer (Pierce) that contained complete Mini protease inhibitor cocktail (Roche), phosphatase inhibitors cocktail (Pierce), 5 mM EDTA, and 1 mM phenylmethylsufonyl fluoride and incubated for 20 min on ice. Cell lysates were prepared by sonication of cells for 1 min on ice. Lysates were centrifuged at 12,000×g for 10 min at 4°C. Preclearing of the lysate was performed by incubation with 50 µl of protein A/G sepharose 50% slurry (Pierce) and 20 µl of normal mouse IgG agarose-conjugated beads (Santa Cruz Biotechnology) for 1 h at 4°C. The lysate was centrifuged briefly, and supernatant was collected. The supernatants containing 500 µg of protein (1 mg/ml) were incubated with 5 µg of either agarose-conjugated mouse anti-pTyr monoclonal antibody or normal mouse IgG with gentle mixing for 16 h at 4°C. The beads were centrifuged briefly for 30 sec at 1,000×g, and then washed three times with lysis buffer and once with PBS before boiling for 5 min in 30 µl of 2x LDS sample buffer with 1x sample reducing agent (Invitrogen). The immunoprecipitated proteins were separated on 4–12% Bis-Tris gel (Invitrogen) by SDS-PAGE and transferred to nitrocellulose membrane. Membrane was blocked in 5% phosphoblocker (Cell Biolabs) in Tris buffered saline with 0.5% Tween (TBST) for 1 h at room temperature. The membranes were then incubated with horseradish peroxidase-conjugated anti-pTyr or rabbit anti-TRP47 antibody. Bound primary antibodies were detected with alkaline phosphatase-conjugated anti-rabbit immunoglobulin G [IgG(H+L)] secondary antibody (Kirkegaard & Perry Laboratories, Gaithersburg, MD) and visualized after incubation with BCIP/NBT (5-bromo-4-chloro-3-indolylphosphate–nitroblue tetrazolium) substrate or with TMB (3,3′,5,5′-tetramethylbenzidine) membrane peroxidase substrate (KPL).

### Chemical Modification of Native and Recombinant TRP47

Native TRP47 was purified from the cell-free supernatant collected from *E. chaffeensis-*infected DH82 cells as previously described [8], except that cell culture supernatants were centrifuged twice (500×g for 5 min and 10,000×g for 10 min) to pellet cells and bacteria. The cell culture supernatants were separated by gel electrophoresis and TRP47 was excised from the gel, electroeluted, concentrated and finally resuspended in 20 mM Tris-HCl (pH 7.6). To 500 µg/ml of GST-only, GST-TRP47, GST-NterTRP47, and GST-CterTRP47 fusion proteins, 0.5 M ethanolamine, 30 mM 2-(*N*-morpholino) ethanesulfonic acid (MES) buffer, and 12 mM 1-ethyl-3-(3-dimethylaminopropyl)carbodiimide (EDC) were added to final concentration, and the reaction mixture was incubated for 1 h at room temperature. The reaction was stopped by addition of LDS sample buffer with sample reducing agent (Invitrogen).

## References

[1] Barnewall RE, Rikihisa Y, Lee EH (1997). *Ehrlichia chaffeensis* inclusions are early endosomes which selectively accumulate transferrin receptor.. Infect Immun.

[2] Lee EH, Rikihisa Y (1998). Protein kinase A-mediated inhibition of gamma interferon-induced tyrosine phosphorylation of Janus kinases and latent cytoplasmic transcription factors in human monocytes by *Ehrlichia chaffeensis*.. Infect Immun.

[3] Lin M, Rikihisa Y (2004). *Ehrlichia chaffeensis* downregulates surface Toll-like receptors 2/4, CD14 and transcription factors PU.1 and inhibits lipopolysaccharide activation of NF-kappa B, ERK 1/2 and p38 MAPK in host monocytes.. Cell Microbiol.

[4] Chen SM, Cullman LC, Walker DH (1997). Western immunoblotting analysis of the antibody responses of patients with human monocytotropic ehrlichiosis to different strains of *Ehrlichia chaffeensis* and *Ehrlichia canis*.. Clin Diagn Lab Immunol.

[5] Chen SM, Dumler JS, Feng HM, Walker DH (1994). Identification of the antigenic constituents of *Ehrlichia chaffeensis*.. Am J Trop Med Hyg.

[6] McBride JW, Corstvet RE, Gaunt SD, Boudreaux C, Guedry T (2003). Kinetics of antibody response to *Ehrlichia canis* immunoreactive proteins.. Infect Immun.

[7] Rikihisa Y, Ewing SA, Fox JC (1994). Western immunoblot analysis of *Ehrlichia chaffeensis*, *E. canis*, or *E. ewingii* infections in dogs and humans.. J Clin Microbiol.

[8] Doyle CK, Nethery KA, Popov VL, McBride JW (2006). Differentially expressed and secreted major immunoreactive protein orthologs of *Ehrlichia canis* and *E. chaffeensis* elicit early antibody responses to epitopes on glycosylated tandem repeats.. Infect Immun.

[9] Luo T, Zhang X, Wakeel A, Popov VL, McBride JW (2008). A variable-length PCR target protein of *Ehrlichia chaffeensis* contains major species-specific antibody epitopes in acidic serine-rich tandem repeats.. Infect Immun.

[10] Yu XJ, Crocquet-Valdes P, Cullman LC, Walker DH (1996). The recombinant 120-kilodalton protein of *Ehrlichia chaffeensis*, a potential diagnostic tool.. J Clin Microbiol.

[11] Jordan P, Snyder LA, Saunders NJ (2003). Diversity in coding tandem repeats in related *Neisseria* spp.. BMC Microbiol.

[12] Shak JR, Dick JJ, Meinersmann RJ, Perez-Perez GI, Blaser MJ (2009). Repeat-associated plasticity in the *Helicobacter pylori* RD gene family.. J Bacteriol.

[13] Clifton DR, Dooley CA, Grieshaber SS, Carabeo RA, Fields KA (2005). Tyrosine phosphorylation of the chlamydial effector protein Tarp is species specific and not required for recruitment of actin.. Infect Immun.

[14] Gaillard JL, Berche P, Frehel C, Gouin E, Cossart P (1991). Entry of *L. monocytogenes* into cells is mediated by internalin, a repeat protein reminiscent of surface antigens from gram-positive cocci.. Cell.

[15] Palmer GH, Brayton KA (2007). Gene conversion is a convergent strategy for pathogen antigenic variation.. Trends Parasitol.

[16] Wren BW (1991). A family of clostridial and streptococcal ligand-binding proteins with conserved C-terminal repeat sequences.. Mol Microbiol.

[17] Wang J, Chen L, Chen F, Zhang X, Zhang Y (2009). A chlamydial type III-secreted effector protein (Tarp) is predominantly recognized by antibodies from humans infected with *Chlamydia trachomatis* and induces protective immunity against upper genital tract pathologies in mice.. Vaccine.

[18] Kling DE, Gravekamp C, Madoff LC, Michel JL (1997). Characterization of two distinct opsonic and protective epitopes within the alpha C protein of the group B Streptococcus.. Infect Immun.

[19] McGarey DJ, Barbet AF, Palmer GH, McGuire TC, Allred DR (1994). Putative adhesins of *Anaplasma marginale*: major surface polypeptides 1a and 1b.. Infect Immun.

[20] Garcia-Garcia JC, de la Fuente J, Bell-Eunice G, Blouin EF, Kocan KM (2004). Glycosylation of *Anaplasma marginale* major surface protein 1a and its putative role in adhesion to tick cells.. Infect Immun.

[21] Popov VL, Yu X, Walker DH (2000). The 120 kDa outer membrane protein of *Ehrlichia chaffeensis*: preferential expression on dense-core cells and gene expression in *Escherichia coli* associated with attachment and entry.. Microb Pathog.

[22] de la Fuente J, Garcia-Garcia JC, Blouin EF, Rodriguez SD, Garcia MA (2001). Evolution and function of tandem repeats in the major surface protein 1a of the ehrlichial pathogen *Anaplasma marginale*.. Anim Health Res Rev.

[23] de la Fuente J, Garcia-Garcia JC, Barbet AF, Blouin EF, Kocan KM (2004). Adhesion of outer membrane proteins containing tandem repeats of *Anaplasma* and *Ehrlichia* species (Rickettsiales: Anaplasmataceae) to tick cells.. Vet Microbiol.

[24] Storey JR, Doros-Richert LA, Gingrich-Baker C, Munroe K, Mather TN (1998). Molecular cloning and sequencing of three granulocytic *Ehrlichia* genes encoding high-molecular-weight immunoreactive proteins.. Infect Immun.

[25] Wakeel A, Kuriakose JA, McBride JW (2009). An *Ehrlichia chaffeensis* tandem repeat protein interacts with multiple host targets involved in cell signaling, transcriptional regulation, and vesicle trafficking.. Infect Immun.

[26] Benz I, Schmidt MA (2002). Never say never again: protein glycosylation in pathogenic bacteria.. Mol Microbiol.

[27] Szymanski CM, Wren BW (2005). Protein glycosylation in bacterial mucosal pathogens.. Nat Rev Microbiol.

[28] Troese MJ, Sarkar M, Galloway NL, Thomas RJ, Kearns SA (2009). Differential expression and glycosylation of *Anaplasma phagocytophilum* major surface protein 2 paralogs during cultivation in sialyl Lewis x-deficient host cells.. Infect Immun.

[29] Upreti RK, Kumar M, Shankar V (2003). Bacterial glycoproteins: functions, biosynthesis and applications.. Proteomics.

[30] Schmidt MA, Riley LW, Benz I (2003). Sweet new world: glycoproteins in bacterial pathogens.. Trends Microbiol.

[31] McBride JW, Yu XJ, Walker DH (2000). Glycosylation of homologous immunodominant proteins of *Ehrlichia chaffeensis* and *Ehrlichia canis*.. Infect Immun.

[32] McBride JW, Doyle CK, Zhang X, Cardenas AM, Popov VL (2007). Identification of a glycosylated *Ehrlichia canis* 19-kilodalton major immunoreactive protein with a species-specific serine-rich glycopeptide epitope.. Infect Immun.

[33] Singu V, Liu H, Cheng C, Ganta RR (2005). *Ehrlichia chaffeensis* expresses macrophage- and tick cell-specific 28-kilodalton outer membrane proteins.. Infect Immun.

[34] Singu V, Peddireddi L, Sirigireddy KR, Cheng C, Munderloh U (2006). Unique macrophage and tick cell-specific protein expression from the p28/p30-outer membrane protein multigene locus in *Ehrlichia chaffeensis* and *Ehrlichia canis*.. Cell Microbiol.

[35] Sarkar M, Troese MJ, Kearns SA, Yang T, Reneer DV (2008). *Anaplasma phagocytophilum* MSP2(P44)-18 predominates and is modified into multiple isoforms in human myeloid cells.. Infect Immun.

[36] Hotopp JC, Lin M, Madupu R, Crabtree J, Angiuoli SV (2006). Comparative genomics of emerging human ehrlichiosis agents.. PLoS Genet.

[37] Mavromatis K, Doyle CK, Lykidis A, Ivanova N, Francino MP (2006). The genome of the obligately intracellular bacterium *Ehrlichia canis* reveals themes of complex membrane structure and immune evasion strategies.. J Bacteriol.

[38] Cantarel BL, Coutinho PM, Rancurel C, Bernard T, Lombard V (2009). The Carbohydrate-Active EnZymes database (CAZy): an expert resource for Glycogenomics.. Nucleic Acids Res.

[39] Stimson E, Virji M, Makepeace K, Dell A, Morris HR (1995). Meningococcal pilin: a glycoprotein substituted with digalactosyl 2,4-diacetamido-2,4,6-trideoxyhexose.. Mol Microbiol.

[40] Hegge FT, Hitchen PG, Aas FE, Kristiansen H, Lovold C (2004). Unique modifications with phosphocholine and phosphoethanolamine define alternate antigenic forms of *Neisseria gonorrhoeae* type IV pili.. Proc Natl Acad Sci U S A.

[41] Schirm M, Soo EC, Aubry AJ, Austin J, Thibault P (2003). Structural, genetic and functional characterization of the flagellin glycosylation process in *Helicobacter pylori*.. Mol Microbiol.

[42] McNally DJ, Aubry AJ, Hui JP, Khieu NH, Whitfield D (2007). Targeted metabolomics analysis of *Campylobacter coli* VC167 reveals legionaminic acid derivatives as novel flagellar glycans.. J Biol Chem.

[43] Voisin S, Kus JV, Houliston S, St-Michael F, Watson D (2007). Glycosylation of *Pseudomonas aeruginosa* strain Pa5196 type IV pilins with mycobacterium-like alpha-1,5-linked d-Araf oligosaccharides.. J Bacteriol.

[44] Luo T, Zhang X, McBride JW (2009). Major species-specific antibody epitopes of the *Ehrlichia chaffeensis* p120 and *E. canis* p140 orthologs in surface-exposed tandem repeat regions.. Clin Vaccine Immunol.

[45] Backert S, Selbach M (2005). Tyrosine-phosphorylated bacterial effector proteins: the enemies within.. Trends Microbiol.

[46] Lin M, den Dulk-Ras A, Hooykaas PJ, Rikihisa Y (2007). *Anaplasma phagocytophilum* AnkA secreted by type IV secretion system is tyrosine phosphorylated by Abl-1 to facilitate infection.. Cell Microbiol.

[47] Mehlitz A, Banhart S, Hess S, Selbach M, Meyer TF (2008). Complex kinase requirements for *Chlamydia trachomatis* Tarp phosphorylation.. FEMS Microbiol Lett.

[48] Jewett TJ, Dooley CA, Mead DJ, Hackstadt T (2008). *Chlamydia trachomatis* tarp is phosphorylated by src family tyrosine kinases.. Biochem Biophys Res Commun.

[49] Selbach M, Paul FE, Brandt S, Guye P, Daumke O (2009). Host cell interactome of tyrosine-phosphorylated bacterial proteins.. Cell Host Microbe.

[50] Grangeasse C, Cozzone AJ, Deutscher J, Mijakovic I (2007). Tyrosine phosphorylation: an emerging regulatory device of bacterial physiology.. Trends Biochem Sci.

[51] Aebersold R, Goodlett DR (2001). Mass spectrometry in proteomics.. Chem Rev.

[52] Graceffa P, Jancso A, Mabuchi K (1992). Modification of acidic residues normalizes sodium dodecyl sulfate-polyacrylamide gel electrophoresis of caldesmon and other proteins that migrate anomalously.. Arch Biochem Biophys.

[53] Armstrong DJ, Roman A (1993). The anomalous electrophoretic behavior of the human papillomavirus type 16 E7 protein is due to the high content of acidic amino acid residues.. Biochem Biophys Res Commun.

[54] Garcia-Ortega L, De los Rios V, Martinez-Ruiz A, Onaderra M, Lacadena J (2005). Anomalous electrophoretic behavior of a very acidic protein: ribonuclease U2.. Electrophoresis.

[55] Alves VS, Pimenta DC, Sattlegger E, Castilho BA (2004). Biophysical characterization of Gir2, a highly acidic protein of *Saccharomyces cerevisiae* with anomalous electrophoretic behavior.. Biochem Biophys Res Commun.

[56] Alves VS, Castilho BA (2005). Gir2 is an intrinsically unstructured protein that is present in *Saccharomyces cerevisiae* as a group of heterogeneously electrophoretic migrating forms.. Biochem Biophys Res Commun.

[57] Sterba J, Vancova M, Rudenko N, Golovchenko M, Tremblay TL (2008). Flagellin and outer surface proteins from *Borrelia burgdorferi* are not glycosylated.. J Bacteriol.

[58] Hu X, Rea HC, Wiktorowicz JE, Perez-Polo JR (2006). Proteomic analysis of hypoxia/ischemia-induced alteration of cortical development and dopamine neurotransmission in neonatal rat.. J Proteome Res.

